# Implementation of the COVID-19 Vulnerability Index Across an International Network of Health Care Data Sets: Collaborative External Validation Study

**DOI:** 10.2196/21547

**Published:** 2021-04-05

**Authors:** Jenna M Reps, Chungsoo Kim, Ross D Williams, Aniek F Markus, Cynthia Yang, Talita Duarte-Salles, Thomas Falconer, Jitendra Jonnagaddala, Andrew Williams, Sergio Fernández-Bertolín, Scott L DuVall, Kristin Kostka, Gowtham Rao, Azza Shoaibi, Anna Ostropolets, Matthew E Spotnitz, Lin Zhang, Paula Casajust, Ewout W Steyerberg, Fredrik Nyberg, Benjamin Skov Kaas-Hansen, Young Hwa Choi, Daniel Morales, Siaw-Teng Liaw, Maria Tereza Fernandes Abrahão, Carlos Areia, Michael E Matheny, Kristine E Lynch, María Aragón, Rae Woong Park, George Hripcsak, Christian G Reich, Marc A Suchard, Seng Chan You, Patrick B Ryan, Daniel Prieto-Alhambra, Peter R Rijnbeek

**Affiliations:** 1 Janssen Research & Development Titusville, NJ United States; 2 Department of Biomedical Sciences Ajou University Graduate School of Medicine Suwon Republic of Korea; 3 Department of Medical Informatics Erasmus University Medical Center Rotterdam Netherlands; 4 Fundacio Institut Universitari per a la recerca a l'Atencio Primaria de Salut Jordi Gol i Gurina Barcelona Spain; 5 Department of Biomedical Informatics Columbia University New York, NY United States; 6 School of Public Health and Community Medicine University of New South Wales Sydney Australia; 7 Tufts Institute for Clinical Research and Health Policy Studies Boston, MA United States; 8 Department of Veterans Affairs University of Utah Salt Lake City, UT United States; 9 Real World Solutions IQVIA Cambridge, MA United States; 10 Melbourne School of Public Health The University of Melbourne Victoria Australia; 11 School of Public Health Peking Union Medical College Beijing China; 12 Department of Real-World Evidence Trial Form Support Barcelona Spain; 13 Department of Public Health Erasmus University Medical Center Rotterdam Netherlands; 14 Department of Biomedical Data Sciences Leiden University Medical Center Leiden Netherlands; 15 School of Public Health and Community Medicine Institute of Medicine Sahlgrenska Academy, University of Gothenburg Gothenburg Sweden; 16 Clinical Pharmacology Unit Zealand University Hospital Roskilde Denmark; 17 NNF Centre for Protein Research University of Copenhagen Copenhagen Denmark; 18 Department of Infectious Diseases Ajou University School of Medicine Suwon Republic of Korea; 19 Division of Population Health and Genomics University of Dundee Dundee United Kingdom; 20 Faculty of Medicine University of Sao Paulo Sao Paulo Brazil; 21 Nuffield Department of Clinical Neurosciences University of Oxford Oxford United Kingdom; 22 Department of Veterans Affairs Vanderbilt University Nashville, TN United States; 23 Department of Biomedical Informatics Ajou University School of Medicine Suwon Republic of Korea; 24 Department of Biostatistics UCLA Fielding School of Public Health University of California Los Angeles, CA United States; 25 Centre for Statistics in Medicine Nuffield Department of Orthopaedics, Rheumatology and Musculoskeletal Sciences University of Oxford Oxford United Kingdom

**Keywords:** external validation, transportability, COVID-19, prognostic model, prediction, C-19, modeling, datasets, observation, hospitalization, bias, risk, decision-making

## Abstract

**Background:**

SARS-CoV-2 is straining health care systems globally. The burden on hospitals during the pandemic could be reduced by implementing prediction models that can discriminate patients who require hospitalization from those who do not. The COVID-19 vulnerability (C-19) index, a model that predicts which patients will be admitted to hospital for treatment of pneumonia or pneumonia proxies, has been developed and proposed as a valuable tool for decision-making during the pandemic. However, the model is at high risk of bias according to the “prediction model risk of bias assessment” criteria, and it has not been externally validated.

**Objective:**

The aim of this study was to externally validate the C-19 index across a range of health care settings to determine how well it broadly predicts hospitalization due to pneumonia in COVID-19 cases.

**Methods:**

We followed the Observational Health Data Sciences and Informatics (OHDSI) framework for external validation to assess the reliability of the C-19 index. We evaluated the model on two different target populations, 41,381 patients who presented with SARS-CoV-2 at an outpatient or emergency department visit and 9,429,285 patients who presented with influenza or related symptoms during an outpatient or emergency department visit, to predict their risk of hospitalization with pneumonia during the following 0-30 days. In total, we validated the model across a network of 14 databases spanning the United States, Europe, Australia, and Asia.

**Results:**

The internal validation performance of the C-19 index had a C statistic of 0.73, and the calibration was not reported by the authors. When we externally validated it by transporting it to SARS-CoV-2 data, the model obtained C statistics of 0.36, 0.53 (0.473-0.584) and 0.56 (0.488-0.636) on Spanish, US, and South Korean data sets, respectively. The calibration was poor, with the model underestimating risk. When validated on 12 data sets containing influenza patients across the OHDSI network, the C statistics ranged between 0.40 and 0.68.

**Conclusions:**

Our results show that the discriminative performance of the C-19 index model is low for influenza cohorts and even worse among patients with COVID-19 in the United States, Spain, and South Korea. These results suggest that C-19 should not be used to aid decision-making during the COVID-19 pandemic. Our findings highlight the importance of performing external validation across a range of settings, especially when a prediction model is being extrapolated to a different population. In the field of prediction, extensive validation is required to create appropriate trust in a model.

## Introduction

### Background

The novel coronavirus SARS-CoV-2, which causes COVID-19, is quickly spreading throughout the world and burdening health care systems worldwide [1]. Numerous prediction models are being developed and released to the public to aid decision-making during the pandemic [2]. Many of these models aim to inform people of their risk of developing severe outcomes due to COVID-19 [3-5]. A recent systematic review found that all the then-published models suffered from high risk of bias along with one or more limitations, including small data sets used to develop the models and lack of external validation [2].

The COVID-19 vulnerability (C-19) index [5] is an example of a prognostic model developed to identify people susceptible to severe outcomes during COVID-19 infection. The model is potentially valuable because it aims to predict hospitalization risk in the general population [2]. At the time of the study, a paper on the model was available as a preprint [5], and the model itself was publicly available at a website [6]. The C-19 index aims to predict which patients will require hospitalization due to pneumonia (or proxies for pneumonia) within 3 months. The model was developed using retrospectively collected Medicare data (patients aged 65 years or older) that did not include patients with COVID-19.

### Objectives

In this paper, we aim to show the importance of external validation and demonstrate the feasibility, during times of urgency, of using a collaborate network for this purpose. We chose to demonstrate this with the C-19 index because it is available as a commercial product to the public, prior to being peer-reviewed, as a model that can predict COVID-19 severity, but it has not undergone any external validation. It is unknown whether this model is currently being used for medical decision-making, but it has been advertised as a decision-making tool. However, the process illustrated in this paper and the lessons learned are applicable to any COVID-19 prediction model. Furthermore, the C-19 index model was developed using non–COVID-19 data, and there is no guarantee that a model trained on Medicare patients who do not have COVID-19 will perform similarly or even adequately in patients with COVID-19. Research has shown that there is high risk of bias for a model that lacks external validation [7]. In addition, it is recommended to assess the knowledge of a model’s reproducibility and transportability before it is used clinically [8]. Models must be reliable, as poor predictions can be detrimental to decision-making [2].

The Observational Health Data Science and Informatics (OHDSI) collaboration is a group of researchers who are collaborating to develop best practices for analyzing observational health care data [9]. OHDSI has developed a framework that enables timely validation of prediction models across a large number of data sets worldwide [10]. The OHDSI network currently contains large COVID-19 cohorts from the United States, Europe, and Asia. In this study, we aim to demonstrate the importance of performing external validation of a model before its predictions can be trusted. As a case study, we chose to investigate the predictive performance of the C-19 index when applied to COVID-19 data from databases across the world. This study provides information about the suitability of using the C-19 index model to aid decision-making during the COVID-19 pandemic.

## Methods

### Existing C-19 Index Models

Three models were developed in the C-19 index paper [5]. The simplest model was a logistic regression with a limited number of predictors: age, sex, hospital usage, 11 comorbidities, and their age interactions. The other two models were less parsimonious gradient boosting machines with more than 500 variables. Only one of these gradient boosting machine models was reported. Withholding a model results in noncompliance with the TRIPOD (Transparent Reporting of a Multivariable Prediction Model for Individual Prognosis or Diagnosis) statement [11] and makes external validation impossible. In this paper, we chose to evaluate the simple logistic regression model, recognizing that COVID-19 prediction models are urgently needed worldwide and that parsimonious models are more readily implemented across health care settings.

### Data Source

Electronic medical records (EMRs) and administrative claims databases from primary care and secondary care systems containing patients from Australia, Japan, the Netherlands, Spain, South Korea, and the United States were analyzed in a distributed network, as detailed in Multimedia Appendix 1, Table S1. Of these data sets, 5 contained COVID-19 cases and 9 did not. All data sets used in this paper were mapped into the OHDSI Observational Medical Outcomes Partnership Common Data Model (OMOP-CDM) [12]. The OMOP-CDM was developed to provide researchers with diverse data sets with a standard database structure. This enables analysis code and software to be shared among researchers, which facilitates external validation of prediction models. Deidentified or pseudonymized data were obtained from routinely collected records from clinical practice. Analyses were performed using the following databases: the Australia Electronic Practice–Based Research Network (AU-ePBRN) (linked primary and secondary care database from Australia); Japanese Medical Data Center (JMDC) (Japanese claims); Integrated Primary Care Information (IPCI) (primary care EMR from the Netherlands); Information System for Research in Primary Care (SIDIAP) (primary care EMR from Spain); Ajou University School of Medicine (AUSOM) and Health Insurance Review and Assessment (HIRA) (EMR and claims database, respectively, from South Korea); Commercial Claims and Encounters (CCAE), ClinFormatics, Medicare (MDCR), Medicaid (MDCD) (US claims databases), Optum EHR, Department of Veterans Affairs (VA), Columbia University Irving Medical Center (CUIMC) and Tufts Medical Center Research Data Warehouse (TRDW) (US EMRs). All analyses were conducted locally in a distributed network in which the analysis code was sent to participating sites and only aggregate summary statistics were returned, with no sharing of patient-level data between organizations.

### Consent to Publish

Each site obtained institutional review board approval for the study or used deidentified data; therefore, the study was determined not to be human subject research. Informed consent was not necessary at any site.

### Participants

The purpose of the C-19 index is to identify which patients with COVID-19 are more likely to require hospitalization due to severe complications. The C-19 index model was developed using non–COVID-19 data; therefore, we externally validated it in (1) COVID-19 cohorts, to see how well the model transports to patients it is being advertised for, and (2) non–COVID-19 cohorts, to see how well the model transports to patients similar to those used to develop it.

We chose to investigate the performance of the model when applied to patients with an outpatient or emergency department (ED) visit with initial symptoms. We chose this approach because it mimics the situation in which patients first seek treatment or medical advice due to developing symptoms or testing positive for COVID-19 (or influenza).

For the external validation using COVID-19 data, patients were included in the target population if they satisfied the criteria below:

- Presenting at an outpatient or ED visit with COVID-19 (COVID-19 was identified by a diagnosis code for SARS-COV-2 or a positive test for SARS-COV-2 that was recorded after January 1, 2020)
- Aged ≥18 years during the outpatient or ED visit
- ≥365 days of observation time in the data prior to the outpatient or ED visit
- No diagnosis of influenza, influenza-like symptoms, or pneumonia in the preceding 60 days (to ensure the index date is the date of the most recent symptom of COVID-19)

The index date was defined as the date of the valid outpatient or ED visit.

For the external validation using non–COVID-19 data (influenza data), patients were included in the target population if they satisfied the criteria below:

- Presenting at an outpatient or ED visit with a record of influenza or influenza-like symptoms (ie, fever and either cough, shortness of breath, myalgia, malaise, or fatigue)
- Aged ≥18 years during the outpatient or ED visit
- ≥365 days of observation time in the data prior to the outpatient/ED visit
- No diagnosis of influenza, influenza-like symptoms, or pneumonia in the preceding 60 days (to ensure the index date is the date of the most recent symptom of influenza)

The index date was defined the date of the valid outpatient or ED visit.

### Outcome

The outcome was hospitalization with pneumonia on the index date (valid outpatient or ED visit) and within 30 days after index.

Multimedia Appendix 2 contains the definitions of pneumonia, influenza, influenza-like symptoms, and COVID-19 used in this study. The full details of the participant cohorts and the outcomes used for validation can be found in the study package [13].

### Predictors

The predictors of the logistic regression version of the C-19 index are age in years, male sex, number of inpatient visits during the prior 12 months, and indicator variables for various Clinical Classifications Software Refined (CCSR) categories. A table with the C-19 predictors and coefficients is presented in Multimedia Appendix 3. The CCSR categories used were pneumonia except that caused by tuberculosis, other and ill-defined heart disease, heart failure, acute rheumatic heart disease, coronary atherosclerosis and other heart disease, pulmonary heart disease, chronic rheumatic heart disease, diabetes mellitus with complication, diabetes mellitus without complication, chronic obstructive pulmonary disease and bronchiectasis, and other specified and unspecified lower respiratory disease. Age interactions with each CCSR variable were also included as predictors. Each CCSR category corresponds to an aggregation of International Classification of Disease, Tenth Revision (ICD-10) codes that belong to the category.

In the development data, if a patient had an ICD-10 code that was part of the CCSR “pneumonia except that caused by tuberculosis” grouping during a specified time period prior to index, their value for the predictor “pneumonia except that caused by tuberculosis” was 1; otherwise, it was 0. This assignment was repeated for each CCSR predictor. Data in the OMOP-CDM do not use ICD-10 codes, but instead use Systematized Nomenclature of Medicine (SNOMED) codes. Therefore, to replicate the predictors in the OMOP-CDM data, we needed to find the sets of SNOMED codes that corresponded to each CCSR predictor. We accomplished this by finding the SNOMED equivalent of each ICD-10 code in a CCSR category.

The SNOMED groupings per CCSR category used by the OHDSI implementation of the C-19 are presented in Multimedia Appendix 3.

### Sample Size

We identified 41,381 patients with an outpatient or ED visit for COVID-19 in 2020: 1985 patients from South Korea, 37,950 patients from Spain, and 1446 patients from the United States. We also identified a total of 9,429,285 patients with an outpatient or ED visit for influenza or influenza-like symptoms in databases from six countries. The number of visits for influenza or influenza-like symptoms per database ranged between 2793 and 3,146,801.

### Missing Data

The prediction models used a cohort design that included any patient who satisfied the inclusion criteria. We did not exclude patients who were lost to follow-up during the 30-day period after the valid outpatient or ED visit.

### Statistical Analysis Methods

The model performance was evaluated using the standard discriminative metrics: area under the receiver operating characteristic (AUROC) curve (equivalent to the C statistic) and area under the precision recall curve (AUPRC). The latter is a useful addition to the AUROC when assessing rare outcomes [14]. An AUROC of 1 corresponds to a model that can always assign a higher risk to patients who will experience the outcome compared to those who will not. An AUROC of 0.5 corresponds to a model that randomly guesses a patient’s risk. Precision is defined as the number of true positives over the number of true positives plus the number of false positives. Recall is defined as the number of true positives over the number of true positives plus the number of false negatives. The precision-recall curve shows the tradeoff between precision and recall for different thresholds. The AUPRC performance is relative to the rareness of the outcome. An AUPRC greater than the percentage of the population with the outcome indicates that the model is discriminating, and the greater the value (closer to 1), the better the discrimination. The AUPRC gives some insight into the false positive rate; a low AUPRC value indicates that the model will lead to many false positives. The calibration was determined by creating deciles based on the predicted risk and plotting the mean predicted risk versus the observed risk in each decile. If a model is well calibrated, the mean predicted risk will be approximately equal to the observed risk for each decile.

We followed the TRIPOD statement guidelines [11] for reporting the model validation throughout this paper. For transparency, an open source package for implementing the model on any OMOP-CDM data is available on GitHub [13].

### Development Versus Validation

The differences between the C-19 index model development settings and the validation settings include a different target population and different data sets. Our validation design settings were chosen to mimic the situation in which a clinician needs to decide whether to admit a patient with COVID-19. Importantly, we validated the C-19 index model on patients with COVID-19.

The C-19 index was developed using a cohort design that entered adult patients into the cohort on September 30, 2016, and predicted whether they would be hospitalized for pneumonia or proxies (influenza, acute bronchitis, or other specified upper respiratory infections) in the following 3 months. Patients were required to have data for 6 or more months, and patients who left the database within 3 months of index and whose deaths were not recorded were excluded. In our external validation, we used a cohort design but entered adult patients into the cohort when they had an initial outpatient/ED visit for influenza (or COVID-19) rather than a fixed date; also, we predicted hospitalization due to pneumonia in 30 days rather than 3 months. We excluded patients with influenza or pneumonia within the 60 days prior to index to restrict the data to initial visits. This mimics the situation during the COVID-19 pandemic in which clinicians need to decide whether to hospitalize a patient initially presenting with COVID-19. We required 12 months of prior observation and did not exclude patients who left the database within 3 months of index.

The C-19 index was developed using a subset of patients from the MDCR database prior to the pandemic. This is a US claims database containing patients aged 65 years or older. In this study, we were able to externally evaluate the C-19 index model on COVID-19 data, including adult patients under 65 years of age, from South Korea, Spain, and the United States.

## Results 

### Web-Based Results

The complete results of our analysis are available as an interactive app [15].

The characteristics of the MDCR data (same data source as the development data but different patient subset) and the HIRA, SIDIAP, and VA data (patients with COVID-19) are displayed in Table 1. The characteristics for all data sets used in the study are available in Multimedia Appendix 4.

**Table 1:** Characteristics of patients at baseline in MDCR (database similar to the development data) and the data sets with COVID-19 data.

Predictor		Target population hospitalization during 30 days after index by data set							
		Medicare supplemental		HIRA^a^		SIDIAP^b^		VA^c^	
		Required	None	Required	None	Required	None	Required	None
Mean age (years)		80.92	76.41	65.53	45.09	63.28	49.61	69.64	58.07
Mean number of inpatient visits in prior 365 days		0.58	0.35	1.38	0.68	—^d^	—	0.32	0.22
Male sex (%)		52	45	56	46	59	43	95	80
**Fraction of patients with a history of each condition (not including index)**									
	Acute rheumatic heart disease	0	0	0	0	—	—	—	—
	Chronic obstructive pulmonary disease and bronchiectasis	0.43	0.25	0.38	0.21	0.06	0.03	0.27	0.21
	Chronic rheumatic heart disease	0.03	0.02	0	0	—	—	—	—
	Coronary atherosclerosis and other heart disease	0.19	0.15	0.21	0.09	0.02	0.01	0.17	0.13
	Diabetes mellitus with complication	0.24	0.18	0.31	0.13	0.03	0.01	0.38	0.24
	Diabetes mellitus without complication	0.38	0.32	0.43	0.20	0.13	0.05	0.50	0.32
	Heart failure	0.37	0.20	0.20	0.07	0.02	0.01	0.23	0.12
	Other and ill-defined heart disease	0.25	0.15	0.02	0.01	0.01	0.01	0.11	0.06
	Other specified and unspecified lower respiratory disease	0.73	0.59	0.92	0.88	0.43	0.38	0.58	0.45
	Pneumonia (except that caused by tuberculosis)	0.39	0.20	0.31	0.15	0.06	0.06	0.20	0.14
	Pulmonary heart disease	0.09	0.04	0.00	0.00	—	—	—	—

^a^HIRA: Health Insurance Review and Assessment.

^b^SIDIAP: Information System for Research in Primary Care.

^c^VA: Department of Veterans Affairs.

^d^—: Data not included due to a low cell count.

### Model Performance

**Figure 1:**
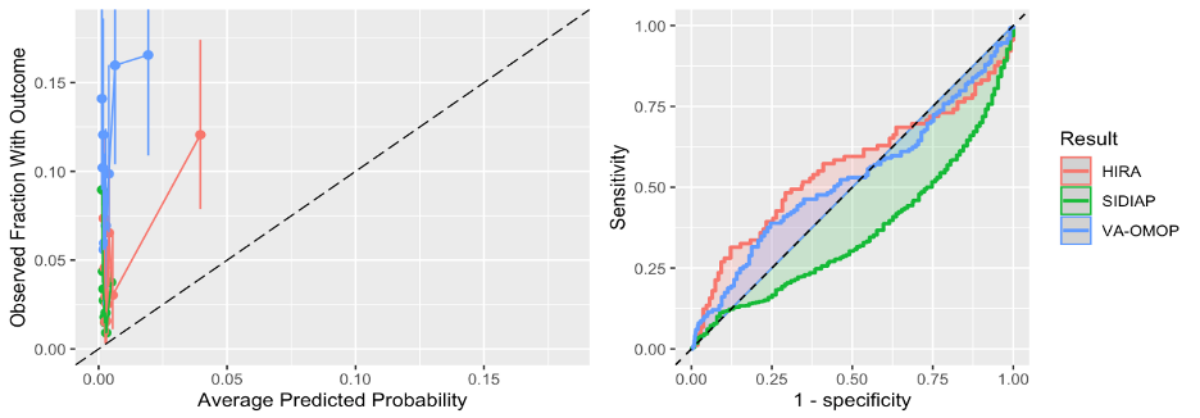
Receiver operating characteristic and calibration plots of the COVID-19 vulnerability index model for the three data sets with sufficient and suitable COVID-19 data. HIRA: Health Insurance Review and Assessment; SIDIAP: Information System for Research in Primary Care; VA-OMOP: Department of Veterans Affairs– Observational Medical Outcomes Partnership.

When C-19 was transported to patients with COVID-19, it achieved AUROCs between 0.36 and 0.56; full details are provided in Table 2. The AUROC and calibration plots are presented in Figure 1. The internal discriminative performance of the C-19 index was an AUROC of 0.73. When we validated the model on patients in the MDCR database (patients aged ≥65 years with supplemental Medicare coverage), but with our target population consisting of symptomatic influenza patients, the performance was 0.65, a substantial drop from the development performance of 0.73. The AUROC performance when externally validated across other databases containing influenza patients ranged between 0.40 and 0.68. Full results are presented in Table 3, and the AUROC and calibration plots are presented in Multimedia Appendix 5. As a sensitivity analysis, we also validated the C-19 index on a target population consisting of patients who had COVID-19 or symptoms of the disease in 2020; the results were similar and are presented in Table S2 in Multimedia Appendix 1.

**Table 2:** External validation of the COVID-19 vulnerability index model on COVID-19 data. The target cohort was patients with an outpatient or emergency department visit with a COVID-19–positive record in 2020 and no symptoms in the prior 60 days.

Database	Target size, n	Outcome size, n (%)	AUROC^a^ (95% CI)^b^	AUPRC^c^
HIRA^d^	1985	89 (4.48)	0.56 (0.488-0.636)	0.07
SIDIAP^e^	37950	1223 (3.22)	0.363	0.03
VA^f^	1446	149 (10.30)	0.529 (0.473-0.584)	0.14

^a^AUROC: area under the receiver operating characteristic curve.

^b^The 95% CI is reported when the outcome count is <1000.

^c^AUPRC: area under the precision recall curve.

^d^HIRA: Health Insurance Review and Assessment.

^e^SIDIAP: Information System for Research in Primary Care.

^f^VA: Department of Veterans Affairs.

**Table 3:** External validation of the COVID-19 vulnerability index model on influenza patient data (non–COVID-19 data).

Database	Target population size, n	Outcome size, n (%)	AUROC^a^ (95% CI)^b^	AUPRC^c^
Medicaid	536,806	32,987 (6.15)	0.68	0.16
Japanese Medical Data Center	1,276,478	728 (0.06)	0.58 (0.55-0.60)	0.004
Medicare supplemental	248,989	31,059 (12.47)	0.65	0.21
Commercial Claims and Encounters	3,146,801	33,824 (1.07)	0.58	0.04
Optum EHR^d^	1,654,157	34,229 (2.07)	0.62	0.07
ClinFormatics	2,082,277	105,030 (5.04)	0.67	0.17
Ajou University School of Medicine	3105	49 (1.58)	0.52 (0.41-0.63)	0.04
Tufts Medical Center Research Data Warehouse	6272	147 (2.34)	0.63 (0.58-0.69)	0.06
Australia Electronic Practice–Based Research Network	2793	29 (1.04)	0.59 (0.45-0.72)	0.03
Columbia University Irving Medical Center	27,356	1121 (5.10)	0.64	0.10
Integrated Primary Care Information	29,132	22 (0.08)	0.40 (0.26-0.54)	0.00
SIDIAP^e^	415,119	512 (0.12)	0.49 (0.45-0.52)	0.00

^a^AUROC: area under the receiver operating characteristic curve.

^b^The 95% CI is reported when the outcome count is <1000.

^c^AUPRC: area under the precision recall curve.

^d^EHR: electronic health record.

^e^SIDIAP: Information System for Research in Primary Care.

## Discussion

The C-19 index is available on the web as a tool to predict severity in patients with COVID-19; however, it lacks validation for this population. Our validation across three data sets with sufficient COVID-19 data showed poor discriminative performance (AUROCs <0.6) and calibration. We observed similarly poor performance when the model was validated across 12 data sets with influenza patients, with the best AUROCs <0.70.

### Interpretation

The key finding of this study is the performance of the C-19 index model when transported to patients with COVID-19. The model performance was poor (AUROCs 0.36-0.56) across the COVID-19 data sets. The performance was worse than random guessing in the SIDIAP data, which is consistent with the poor performance seen when the model was applied to European patients with influenza. The calibration plots show that the C-19 index consistently underestimated risk in the patients with COVID-19.

The data sets used to perform the validation had very different patient populations. MDCR had the oldest patient population, and many patients in this data set had comorbidities. Compared to MDCR, the CCAE and JMDC data sets presented healthier and younger patients (mean age approximately 40 years) in the target population. Although the MDCD data set contained younger patients, these patients often had comorbidities (ie, 20% these patients had chronic obstructive pulmonary disease, 11% had heart failure, and 17% had a history of pneumonia). The rate of hospitalization ranged greatly across the data sets, with values between 0.1% (JMDC) and 12.4% (MDCR). The rate of the outcome in the data set used to develop the C-19 index was 0.23%, much lower than that in the MDCR data set used to validate the model in this study. This is because our study was restricted to patients at the point they had an outpatient or ED visit due to influenza or COVID-19. Although five data sets contained patients with COVID-19, only four (VA, HIRA, SIDIAP, and CUIMC) contained sufficient data for external validation. The result of the C-19 index model when applied to patients with COVID-19 in CUIMC was poor, with an AUROC <0.5; however, this data set consisted mostly of hospitalized patients and therefore did not seem to be suitable for validating a model that predicts hospitalizations.

We chose a target population of symptomatic patients because this resembles the situation in which COVID-19 prediction models may be clinically implemented during the pandemic: clinicians would not be likely to admit asymptomatic patients. This suggests that the internal C-19 AUROC estimate, which was evaluated within the general population rather than among people with symptoms, may be optimistic compared to its use in a realistic setting due to the inclusion of many healthy patients in the model development data. When applied to predict hospitalization in influenza patients across US data, the discriminative performance ranged between 0.58 and 0.68**.** The performance was worse on the CCAE data set with younger patients, likely because age is a key predictor in the model. When the C-19 index was transported across non-US data sets, the discrimination was poor to reasonable in the Australian and Asian data (0.52-0.64) and poor in the European data (0.4-0.49). The European data were extracted from general practice settings, but the C-19 index model was developed using US claims data. Given the differences in clinical settings, it is not surprising that the performance was poor. This finding highlights that models often may not transport to different health care settings. The AUROC of 0.36 when the C-19 index model was validated in SIDIAP was worse than random guessing, and inverting the predicted risk would lead to an AUROC of 0.64. This may be a result of the C-19 including age interaction terms, which resulted in a negative age coefficient. Table 1 shows that in SIDIAP, the model’s age-interacting comorbidities are not recorded as often as in the other databases. As a result, younger patients may have been assigned higher risks than older patients in SIDIAP.

The calibration was poor when applying the C-19 to COVID-19 data. This is not unexpected, as it is known that patients with COVID-19 have a higher risk of hospitalization due to pneumonia than the general COVID-19–free population. The calibration could likely be improved by performing recalibration using a sample of data from patients with COVID-19.

### Implications

The results provide extensive insight into the performance of the logistic regression C-19 index when used for COVID-19 data. The external validation uncovered that the logistic regression C-19 index model is unreliable when predicting hospitalization risk for patients with COVID-19. Given this result, we do not recommend using the logistic regression C-19 index to aid decision-making during the COVID-19 pandemic. The model did not appear to transport to patients with COVID-19, highlighting the importance of externally validating models, especially models whose target population differs from the development population.

There are numerous potential reasons why the logistic regression C-19 index model failed to predict hospitalization due to pneumonia in the investigated patients with COVID-19. The first reason may be that the model was developed on patients aged 65 years or older but was applied to patients aged 18 or older. Age had a negative coefficient in the model, which may have caused issues when the model was applied to younger patients. A second reason may be due to incorrect phenotyping of the predictors. We matched the SNOMED codes to the CCSR ICD-10 codes provided; however, the predictors may require database-specific phenotypes due to coding differences across data sets and health care settings. This may explain the poor performance in the European data sets, which were obtained from databases that may record entries differently than those in the United States. A third reason is the study design. The C-19 index was developed to predict hospitalization from a set date in 2016; however, we validated it in a target cohort of symptomatic patients with an outpatient or ED visit, as this more closely matches the clinical use case of the model. Therefore, we were likely to have a sicker population, in which discrimination may have been more difficult. A fourth potential reason is that the C-19 index model was developed using data prior to 2017 but was validated on data from 2020: temporal changes and concept drift may have negatively impacted the performance. Although we do not know the reason for the unreliability of the C-19 index model on patients with COVID-19, we were able to quantify it by large-scale external validation across a network of data sets. In future work, it would be beneficial to develop techniques that can identify reasons for poor external validation performance, as this may inform new best practices for model development.

This study highlights the importance of performing extensive external validation across different settings. During times of uncertainty, such as pandemics, medical staff who are under pressure to make important decisions could benefit from implementing vetted prediction models. However, it is important to gain an unbiased and reliable evaluation of a model’s performance across numerous patient populations before the model is used. Internal validation can often be biased (eg, the population used to develop the model does not match the intended target population) and can provide optimistic performance estimates (eg, a poor design or small data set may result in overestimated discriminative performance). The approach used by the OHDSI collaboration enables efficient external validation of models across multiple data sets, and this is a valuable resource when urgency is required.

### Limitations

A common issue when using observational health care data, especially across a network of databases, is the difficulty in developing phenotypes that are valid on all data sets. In this study, we used predictor definitions given by the researchers who developed the model. However, these definitions may not transport across all the data sets and may account for some of the decrease in performance. We were also limited to validating the less complex C-19 index model due to the large number of variables and lack of transparency for the more complex models.

The C-19 index model used in this paper to demonstrate the importance of external validation may have limited use for medical decision-making. Other COVID-19 models, such as those including physiological measurements, may be making more clinical impact. However, we choose the C-19 index model because it was available early in the pandemic and was being advertised to the public as a useful tool while being reported in a preprint paper with no formal peer review.

### Conclusions

We have demonstrated the importance of implementing external validation in multiple data sets to determine the reliability of prediction models. We picked a newly developed model, the C-19 index, that aimed to predict which patients with COVID-19 are at risk of severe complications due to SARS-CoV-2. The model reported an internal AUC of 0.73 but was deemed to have a high risk of potential bias [2]. The C-19 index addresses an important issue that could have greatly aided decision-making during the COVID-19 pandemic; however, its performance in patients with COVID-19 was unknown. Our results show that the C-19 index performs poorly when applied to newly diagnosed patients with COVID-19 in Asia, Europe, and the United States. Overall, we suggest that the model currently only be used to predict hospitalization due to pneumonia in older patients in the United States. The results of this study demonstrate that internal validation performance should be considered optimistic estimates and that a prediction model requires validation across multiple data sets in the target population where it will be used (or a close proxy) before it should be trusted.

## Appendix

Multimedia Appendix 1 Supplemental tables describing the databases used in this study and sensitivity results.

Multimedia Appendix 2 Code set used to define each condition.

Multimedia Appendix 3 COVID-19 vulnerability index model and Systematized Nomenclature of Medicine phenotype codes.

Multimedia Appendix 4 Descriptive table for each of the non–COVID-19 case databases.

Multimedia Appendix 5 Receiver operating characteristic and calibration plots for the non–COVID-19 case databases.

## Abbreviations

AU-ePBRN: Australia Electronic Practice–Based Research Network
AUPRC: area under the precision recall curve
AUROC: Area under the receiver operating characteristic curve
AUSOM: Ajou University School of Medicine
C-19: COVID-19 vulnerability
CCAE: Commercial Claims and Encounters
CCSR: Clinical Classifications Software Refined
CUIMC: Columbia University Irving Medical Center
ED: emergency department
EHDEN: European Health Data and Evidence Network
EMR: electronic medical record
HIRA: Health Insurance Review and Assessment
ICD-10: International Classification of Disease, Tenth Revision
IPCI: Integrated Primary Care Information
JU: Joint Undertaking
MDCD: Medicaid
MDCR: Medicare
NIHR: National Institute for Health Research
OHDSI: Observational Health Data Science and Informatics
OMOP-CDM: Observational Medical Outcomes Partnership Common Data Model
SIDIAP: Information System for Research in Primary Care
SNOMED: Systematized Nomenclature of Medicine
TRDW: Tufts Medical Center Research Data Warehouse
TRIPOD: Transparent Reporting of a Multivariable Prediction Model for Individual Prognosis Or Diagnosis
VA: Veterans Affairs
