# Supplementary material for: Implementation of the COVID-19 Vulnerability Index Across an International Network of Health Care Data Sets: Collaborative External Validation Study
Source: JMIR Med Inform. 2021 Apr 5;9(4):e21547. doi: 10.2196/21547 (PMC8023380; doi:10.2196/21547)
Supplement: Multimedia Appendix 1 [file medinform_v9i4e21547_app1.docx]

**Appendix**

*Table S1 Data sources formatted to the OMOP-CDM used in this research*

| **Database** | **Database**  **Acronym** | **Country** | **Data type** | **Contains COVID-19 data?** | **Time period** |
| --- | --- | --- | --- | --- | --- |
| IBM MarketScan® Medicare Supplemental Database | MDCR | US | Claims | No | 2000-2018 |
| Columbia University Irving Medical Center Data Warehouse | CUIMC | US | EMR | Yes | Influenza: 1990-2020  COVID-19: March-April 2020 |
| Health Insurance and Review Assessment | HIRA | South Korea | Claims | Yes | COVID-19: January to April 2020 |
| The Information System for Research in Primary Care | SIDIAP | Spain | GP and hospital admission EHRs linked | Yes | Influenza: 2006-2017  COVID-19: March 2020 |
| Tufts Research Data Warehouse | TRDW | US | EMR | Yes | Influenza: 2006-2020  COVID-19: March 2020 |
| Department of Veterans Affairs | VA | US | EHR | Yes | COVID: 1^st^ March – 20 April 2020 |
| Ajou University School of Medicine Database | AUSOM | South Korea | EHR | No | 1996 - 2018 |
| Australian Electronic Practice based Research Network | AU-ePBRN | Australia | GP and hospital admission EHRs linked | No | 2012-2019 |
| IBM MarketScan® Commercial Database | CCAE | US | Claims | No | 2000-2018 |
| Integrated Primary Care Information | IPCI | Netherlands | GP | Yes | 2006-2020 |
| Japan Medical Data Center | JMDC | Japan | Claims | No | 2005-2018 |
| IBM MarketScan® Multi-State Medicaid Database | MDCD | US | Claims | No | 2006-2017 |
| Optum© De-Identified Clinformatics® Data Mart Database | ClinFormatics | US | Claims | No | 2000-2018 |
| Optum^©^ de-identified Electronic Health Record Dataset | Optum EHR | US | Claims | No | 2006-2018 |

*Table S2 Sensitivity analysis - results with more sensitive but less specific definition for the target population*

| **Database** | **Sensitivity Target Population** | **Target Population size** | **Outcome size (%)** | **AUROC** | **AUPRC** |
| --- | --- | --- | --- | --- | --- |
| HIRA | COVID-19 positive test or symptoms in 2020 | 47,594 | 2,463 (5.18) | 0.64 | 0.1 |
| HIRA | COVID-19 positive test in 2020 | 1,985 | 89 (4.48) | 0.56 | 0.07 |
| TRDW | COVID-19 positive test or symptoms in 2020 | 285 | 5 (1.75) | 0.74 | 0.04 |
| SIDIAP | COVID-19 positive test or symptoms in 2020 | 38,254 | 1,229 (3.21) | 0.366 | 0.03 |
| SIDIAP | COVID-19 positive test in 2020 | 37,950 | 1,223 (3.22) | 0.363 | 0.03 |
| VA | COVID-19 positive test or symptoms in 2020 | 5,990 | 486 (8.11) | 0.627 | 0.15 |
| VA | COVID-19 positive test in 2020 | 1,446 | 149 (10.30) | 0.529 | 0.14 |
