# Supplementary material for: Implementation of the COVID-19 Vulnerability Index Across an International Network of Health Care Data Sets: Collaborative External Validation Study
Source: JMIR Med Inform. 2021 Apr 5;9(4):e21547. doi: 10.2196/21547 (PMC8023380; doi:10.2196/21547)
Supplement: Multimedia Appendix 3 [file medinform_v9i4e21547_app3.docx]

## Appendix B: Predictor SNOMED codes

| Coefficient | Predictor | Snomed codes |
| --- | --- | --- |
| -5.49 | Intercept | NA |
| -0.014 | Age | NA |
| -0.212 | Male | 8507 |
| 0.186 | Number of inpatient visits in last year |  |
| -0.008 | Acute rheumatic heart disease | 313500,321578,321586,437897,4225090,4271684,4306131 |
| -0.269 | Chronic obstructive pulmonary disease and bronchiectasis | 255573,255841,256448,257004,257905,258780,259043,261325,261889,261895,440748,765431,4046986,4050731,4050732,4050733,4050734,4050961,4056405,4083395,4104506,4110048,4110049,4110056,4110635,4112826,4112828,4112836,4115044,4136683,4137525,4138392,4145496,4148124,4163244,4166508,4166517,4172303,4177944,4193588,4196712,4209097,4230358,4246105,4278831,4286497,4309350,4315386,37206130,40481763,42536541,42539089,42574216,42598711,43530693,44791725,44807895,45769389,46269701,46270376,46274062 |
| -0.014 | Chronic rheumatic heart disease | 313221,313500,315282,315295,317296,318776,319825,320429,320743,321578,321586,380126,435829,437897,439834,4006165,4040820,4057759,4062117,4078214,4108085,4108168,4117862,4122085,4143607,4154293,4155486,4156119,4169568,4175807,4178585,4181949,4182110,4184497,4192358,4195677,4209569,4215619,4221806,4222174,4225090,4227150,4231452,4238914,4240289,4244437,4259295,4263730,4264740,4271684,4301373,4304541,4306131,4312621,4313828,4339544,43020465,43020466 |
| -0.529 | Coronary atherosclerosis and other heart disease | 315296,315830,315832,321318,761735,4068938,4078531,4108670,4116486,4119455,4119942,4127089,4155008,4155009,4155963,4161456,4161457,4161973,4161974,4184827,4198141,4201629,4231426,4262446,4264145,4310270,4324893,35615052,35615053,36712982,36712983,36712984,37209632,43531588 |
| -0.273 | Diabetes mellitus with complication | 192279,200687,201530,201531,318712,321822,376065,376112,376114,376683,376979,377552,377821,378743,380096,380097,435216,439770,442793,443592,443727,443729,443730,443731,443732,443733,443734,443735,443767,760977,760978,760979,760980,760989,761048,761050,761051,761053,761062,761063,765373,765375,765533,765650,4007943,4009303,4016047,4023792,4027121,4029420,4029423,4030664,4033942,4034964,4044391,4044392,4044393,4046332,4048028,4048029,4054812,4061725,4063569,4065354,4082346,4082347,4082348,4087682,4095288,4099216,4099652,4101478,4101887,4101892,4102176,4105016,4105172,4105173,4105639,4114426,4114427,4128221,4129225,4129520,4131117,4131908,4137220,4140466,4142579,4143689,4143857,4145542,4147406,4147504,4147577,4147719,4151453,4151946,4159742,4161670,4161671,4162095,4162239,4164174,4164175,4164176,4164632,4169240,4171406,4174977,4175440,4176925,4177050,4189418,4191611,4192852,4194970,4195043,4195044,4195045,4195498,4199039,4200875,4206115,4209538,4210128,4210129,4210872,4210874,4212441,4215719,4215961,4218499,4221344,4221487,4221495,4221933,4221962,4222415,4222553,4222687,4222876,4223303,4223463,4223734,4223739,4224254,4224419,4224709,4224879,4225055,4225656,4226121,4226238,4226354,4226798,4227210,4227657,4228112,4228443,4234742,4235260,4242528,4243625,4247107,4252356,4255399,4255400,4255401,4262282,4263090,4265913,4266041,4266042,4266637,4269870,4269871,4270049,4290822,4290823,4295011,4304701,4307319,4307799,4311708,4321756,4334884,4336000,4338900,4338901,35625717,35625718,35625719,35625722,35625723,35625724,35626036,35626037,35626038,35626039,35626041,35626042,35626043,35626044,35626046,35626047,35626067,35626068,35626069,35626070,35626071,35626072,35626087,35626088,35626761,35626762,35626763,35626764,35626765,35626904,35626905,36674199,36674200,36674651,36674652,36674752,36674753,36674765,36674766,36684827,36712670,36712686,36712687,36713094,36714116,36715571,36716853,36717156,37016179,37016180,37016348,37016349,37016350,37016353,37016354,37016355,37016356,37016357,37016358,37016767,37016768,37017221,37017429,37017430,37017431,37017432,37018566,37018728,37018912,37109305,37110068,37110593,37396268,40480000,40480031,40482458,42535539,42536400,42536603,42536604,42536605,42538169,42538715,43530656,43530660,43530685,43530689,43530690,43531559,43531562,43531563,43531564,43531565,43531566,43531577,43531578,43531588,43531597,43531608,43531616,43531651,43531653,44789318,44789319,44805212,44805628,44809809,45757065,45757073,45757074,45757075,45757255,45757266,45757277,45757278,45757280,45757362,45757363,45757392,45757393,45757432,45757435,45757444,45757445,45757446,45757447,45757449,45757450,45757499,45757507,45757535,45757604,45763582,45763583,45763584,45763585,45766963,45769828,45769829,45769830,45769832,45769833,45769834,45769835,45769836,45769837,45769872,45769873,45769875,45769876,45769888,45769889,45769890,45769891,45769892,45769894,45769901,45769902,45769903,45769904,45769905,45769906,45770830,45770831,45770832,45770880,45770881,45770883,45770902,45770928,45771064,45771067,45771068,45771072,45771075,45771533,45772019,45772060,45772914,45773064,45773567,45773576,45773688,46269764,46274058 |
| -0.496 | Diabetes mellitus without complication | 443412,4008576,4193704,45757474 |
| -0.271 | Heart failure | 312927,314378,315295,316139,316994,319835,439694,439696,439698,439846,442310,443580,443587,444031,444101,762002,762003,764871,764872,764873,764874,764876,764877,4004279,4009047,4014159,4023479,4030258,4071869,4079296,4079695,4103448,4108244,4108245,4111554,4124705,4134890,4138307,4139864,4141124,4142561,4172864,4177493,4184497,4185565,4193236,4195785,4195892,4199500,4205558,4206009,4215446,4215802,4229440,4233224,4233424,4242669,4259490,4264636,4267800,4273632,4284562,4307356,4311437,4327205,35615055,36712927,36712928,36712929,36713488,36716182,36716748,36717359,37110330,40479192,40479576,40480602,40480603,40481042,40481043,40482857,40486933,42598803,43020421,43020657,43021735,43021736,43021825,43021826,43021840,43021841,43021842,43022054,43022068,43530642,43530643,43530961,44782428,44782655,44782713,44782718,44782719,44782728,44782733,44784345,44784442,45766164,45766165,45766166,45766167,45766964,45773075 |
| -0.111 | Other and ill-defined heart disease | 4131824,43021898,40483752,4317287,4069185,42536628,40489421,43022035,4108352,4237062,4100397,4216844,35615119,40483223,40481472,36712751,40487039,4120089,4068741,43020564,43021610,43021955,42594384,42594383,42536629,4173820,4119953,35622329,438171,43021897,4273462,4100132,42599748,4170062,4175580,43021066,37109910,4101319,4182190,4236169,43020636,43020927,4119606,36712752,4033322,36716866,4321717,4100871,40479589,43021064,43020641,4119462,42536642,43021734,36712838,40487573,36712985,42534988,321320,42536633,43020582,42537536,4108220,4108219,4108722,43021065,438168,316427,4102852,4148905,43020889,43021891,4108950,314658,432937,4141491 |
| -0.02 | Other specified and unspecified lower respiratory disease | 4027553 plus all descendants |
| 0.117 | Pneumonia (except caused tuberculosis) | 252351,252548,252655,252949,253235,253790,254066,254561,254677,255084,255735,255848,256722,256723,257315,257908,258061,258180,258333,258354,258785,259048,259852,259992,260028,260041,260430,260754,261053,261324,261326,436145,437313,439857,440431,442637,443410,759815,759816,759817,759818,759821,763011,763012,4021760,4025165,4044215,4045227,4046011,4048052,4048147,4048148,4048149,4048517,4048518,4048519,4049965,4050872,4050874,4051333,4051334,4051335,4051337,4051338,4051339,4052546,4052547,4052548,4070540,4071610,4071611,4080435,4080753,4080883,4082065,4084973,4102253,4110039,4110506,4110507,4110509,4110510,4111119,4112655,4112820,4112822,4114030,4114031,4116487,4116488,4117114,4119431,4119436,4119795,4124539,4133224,4135197,4137435,4138244,4138769,4140134,4141619,4143092,4144107,4145369,4148529,4153356,4166072,4169796,4174308,4174309,4175598,4177385,4186568,4190647,4193964,4195014,4195452,4200891,4203846,4204819,4205578,4212120,4215807,4221503,4221767,4222062,4223032,4225318,4228277,4233319,4236311,4240452,4245006,4245499,4248029,4248154,4248807,4256236,4256894,4267135,4273378,4274802,4274981,4276663,4280213,4284985,4293463,4294404,4299862,4308451,4309106,4310964,4311555,4322625,4327820,4334649,4341520,4345215,4345699,35622404,36676238,36714118,37016927,37017277,37017278,37019058,37110291,37110292,37116366,37119233,37394479,40479642,40480033,40481335,40481839,40482061,42572644,42572881,42573020,42573178,42573179,42573181,42573218,42573349,42593423,42598655,42598908,42598979,42598991,42599060,42599199,42600053,42600167,43020558,44782989,45757206,45757250,45757644,45763749,45763750,45763751,45763752,45767051,45768914,45768960,45768961,45768997,45768998,45769390,45769809,45770900,45771022,46269693,46269707,46269708,46269709,46269710,46269711,46269712,46269713,46269714,46269715,46269716,46269717,46269718,46269719,46269720,46269721,46269722,46269723,46269724,46269725,46269726,46269954,46270027,46270121,46270318,46270510,46274035 |
| -0.005 | Pulmonary heart disease | 312927,315831,317000,433783,441593,4013643,4108610,4119611,4121462,4121620,4124831,4149211,4167085,4195892,4284110,35615055,36715093,40482858,40493243,42536630,42536631,44782560,44782561,44782562,44783618,44783619,44783620,44783621,44783622,44783623,44783624,44783625,44783626,45766142 |
| 0.003 | Acute rheumatic heart disease  X Age | See above |
| 0.013 | Chronic obstructive pulmonary disease and bronchiectasis  X Age | See above |
| -0.001 | Chronic rheumatic heart disease  X Age | See above |
| 0.011 | Coronary atherosclerosis and other heart disease  X Age | See above |
| 0.007 | Diabetes mellitus with complication  X Age | See above |
| 0.009 | Diabetes mellitus without complication  X Age | See above |
| 0.009 | Heart failure  X Age | See above |
| 0.003 | Other and ill-defined heart disease  X Age | See above |
| 0.006 | Other specified and unspecified lower respiratory disease  X Age | See above |
| 0.01 | Pneumonia (except caused tuberculosis)  X Age | See above |
| 0 | Pulmonary heart disease  X Age | See above |
