# Supplementary material for: Implementation of the COVID-19 Vulnerability Index Across an International Network of Health Care Data Sets: Collaborative External Validation Study
Source: JMIR Med Inform. 2021 Apr 5;9(4):e21547. doi: 10.2196/21547 (PMC8023380; doi:10.2196/21547)
Supplement: Multimedia Appendix 5 [file medinform_v9i4e21547_app5.docx]

## Appendix D: ROC and calibration plots

Full results are available from http://evidence.ohdsi.org/C19validation

Plots using Target population of patients with influenza or influenza-like symptoms

| ClinFormatics | | |
| --- | --- | --- |
| 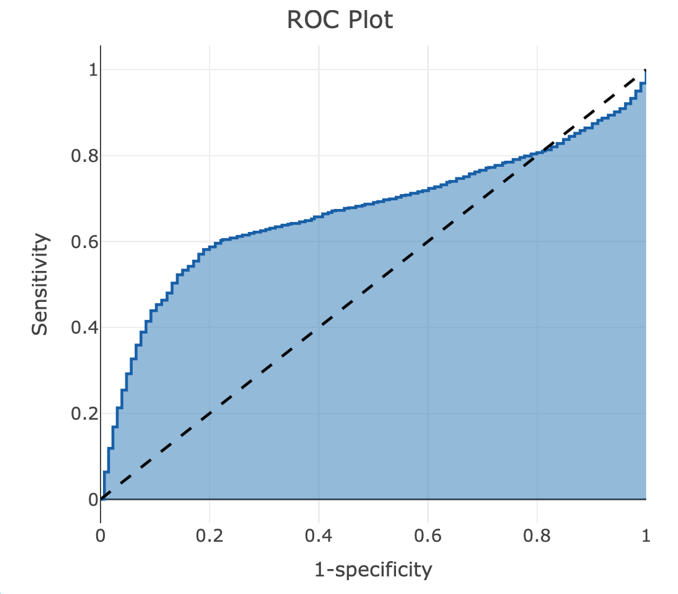 | 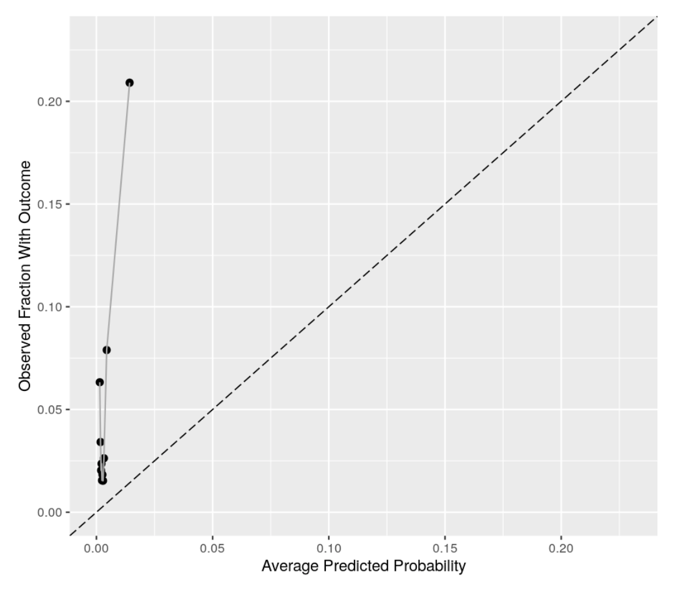 | |
| CCAE | | |
| 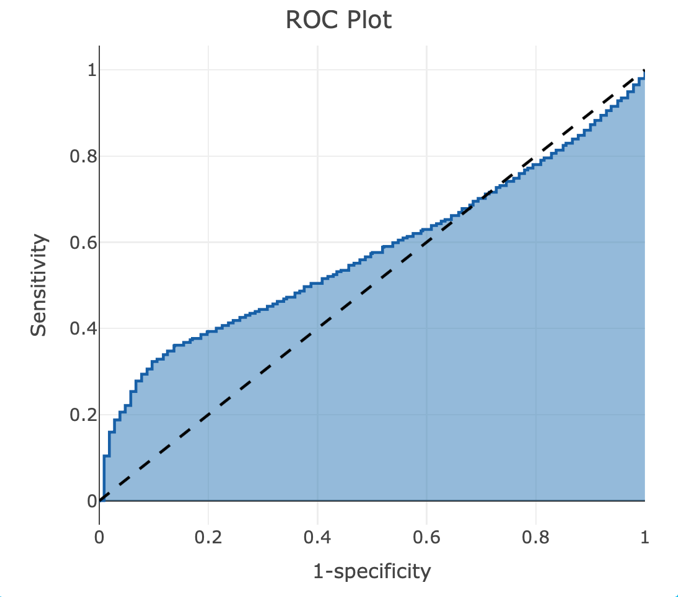 | 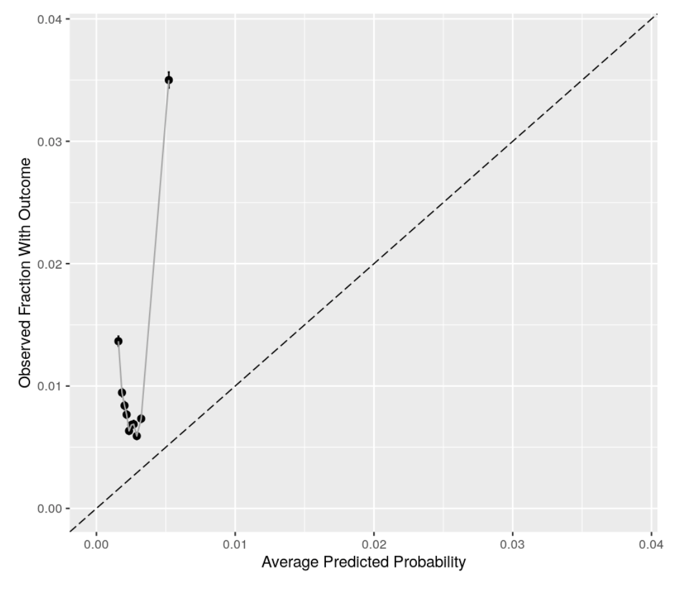 | |
| MDCD | | |
| 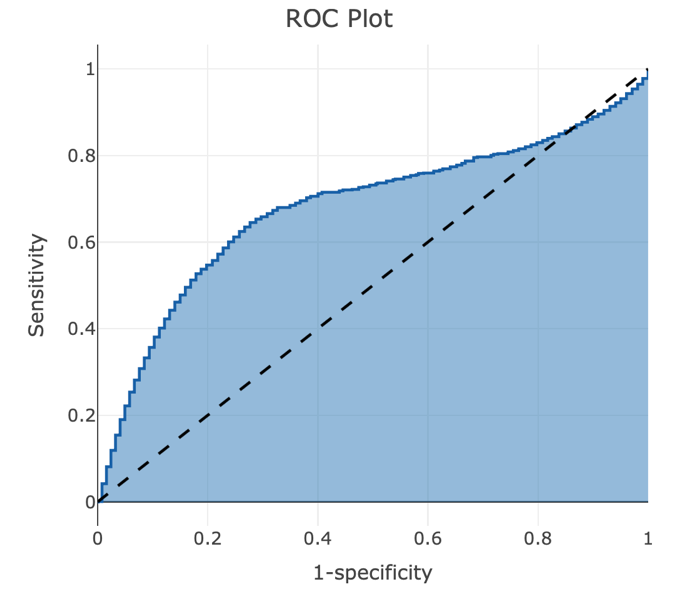 | 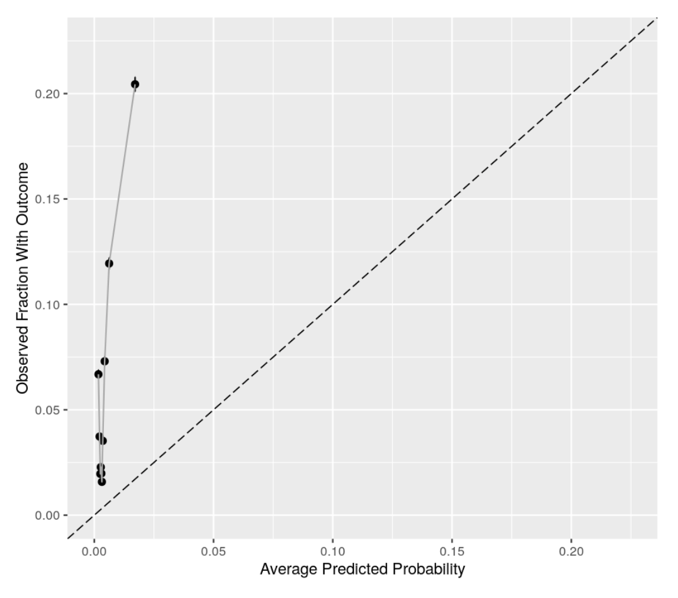 | |
| MDCR | | |
| 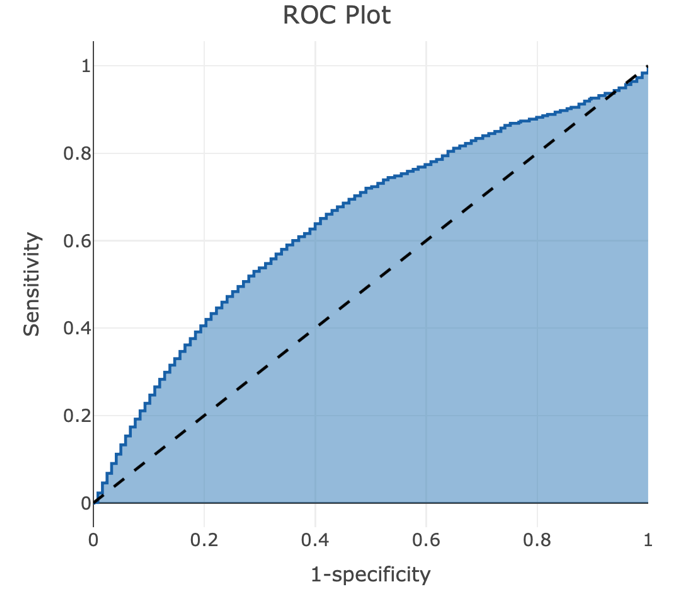 | 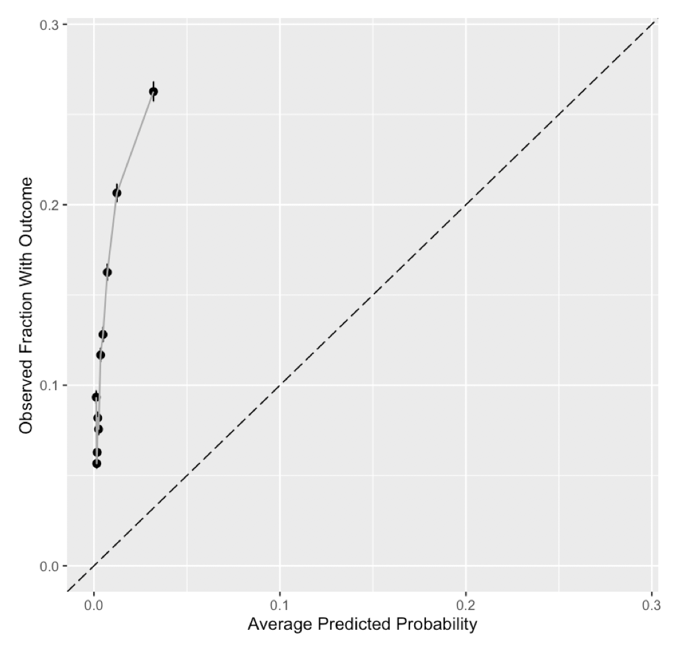 | |
| Optum EHR | | |
| 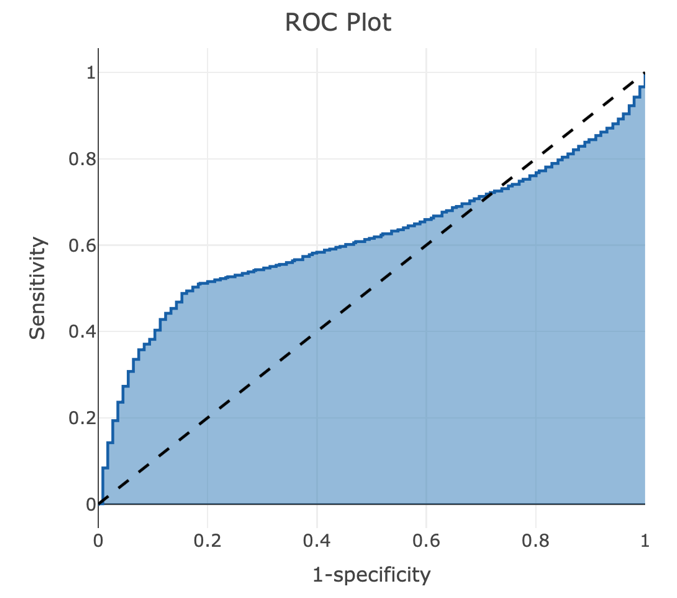 | 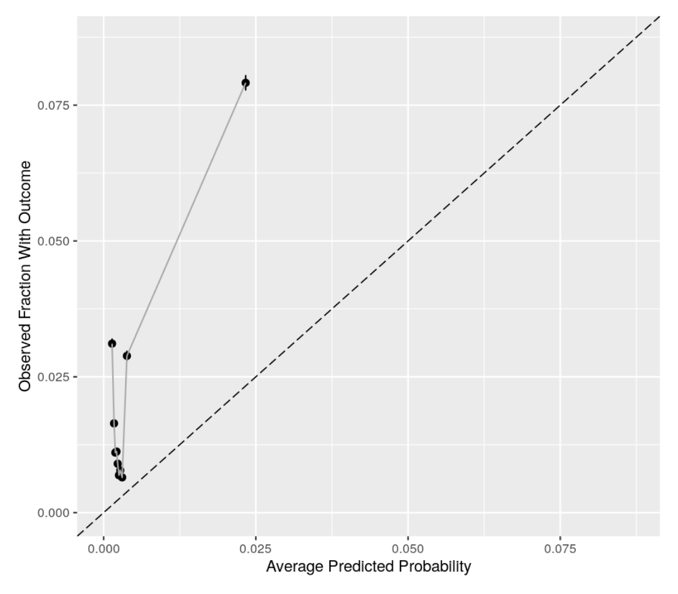 | |
| JMDC | | |
| 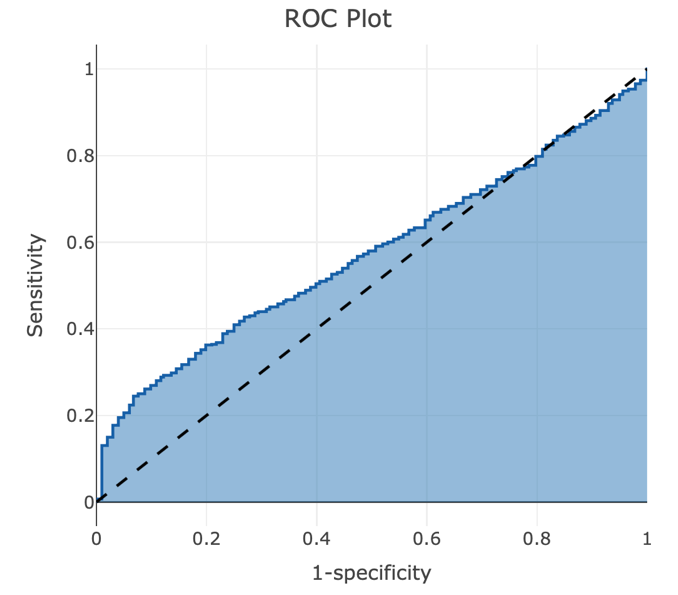 | 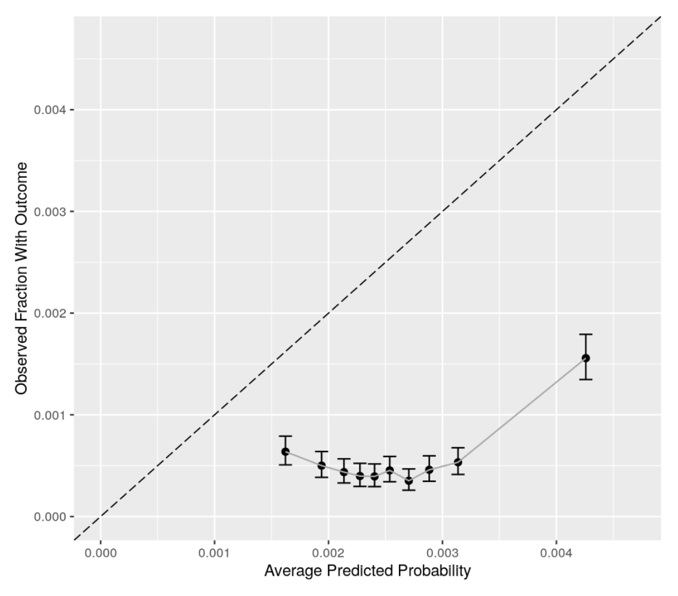 | |
| AUSOM | |  |
| 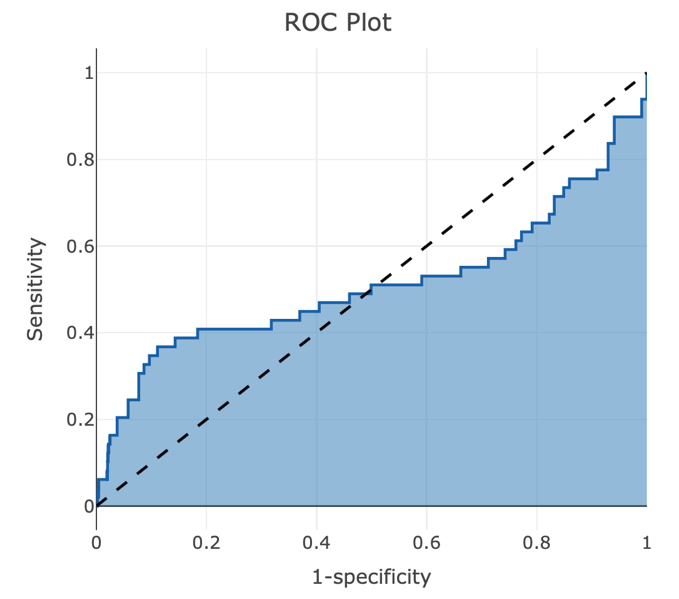 | 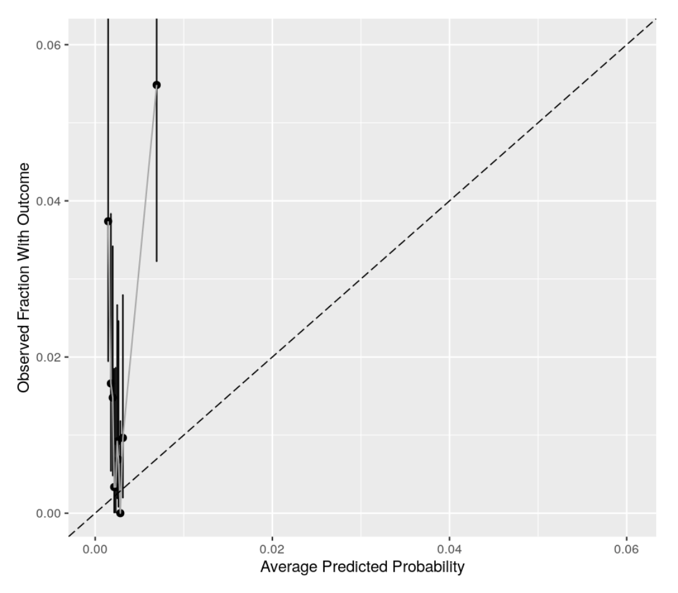 | |
| CUIMC | |  |
| 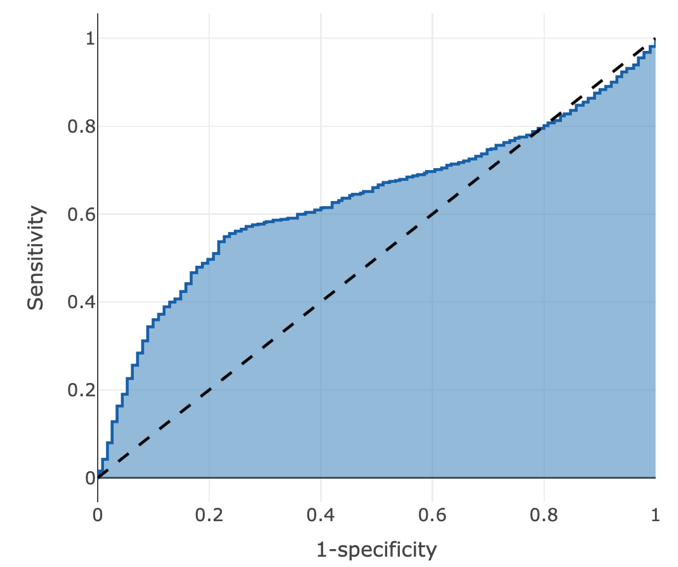 | 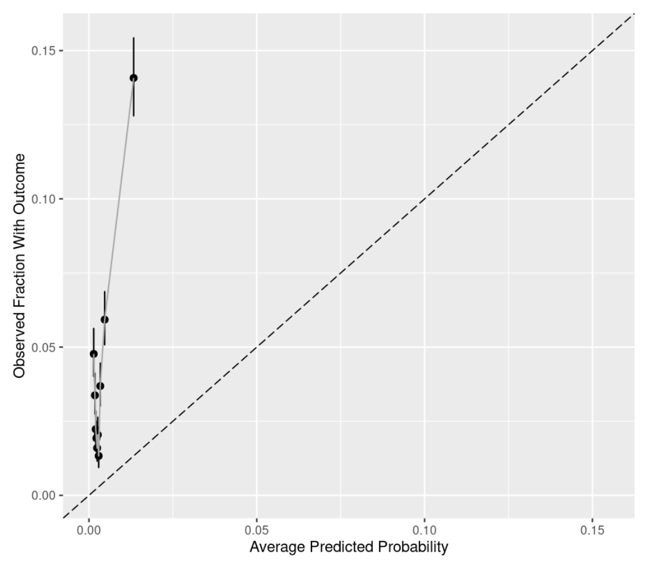 | |
| SIDIAP | | |
| 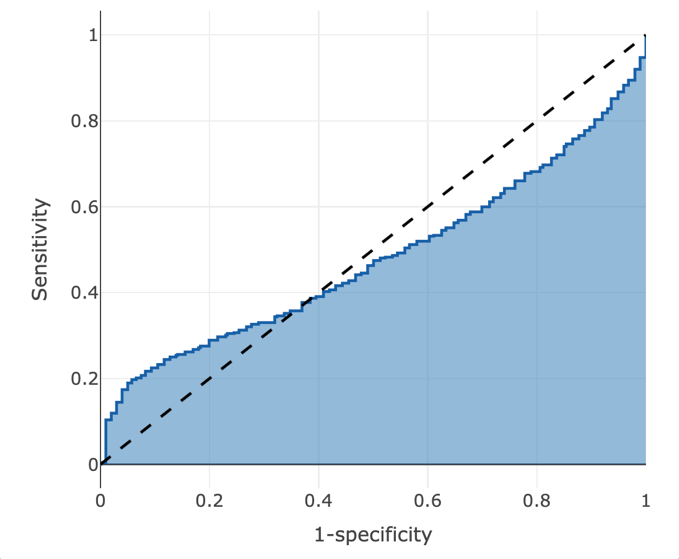 | 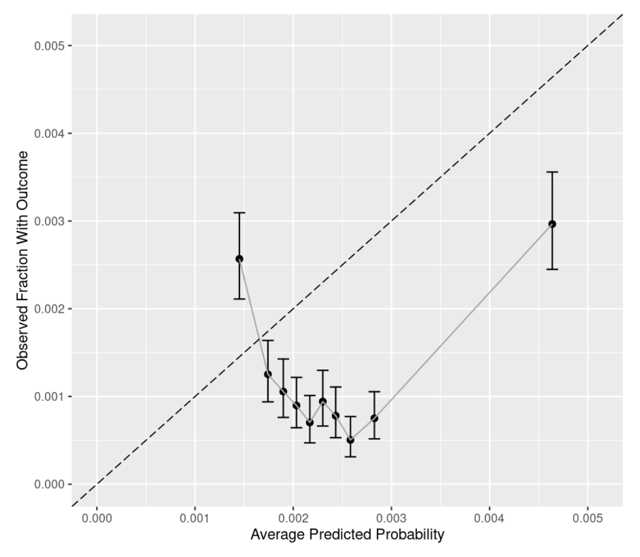 | |
| IPCI | | |
| 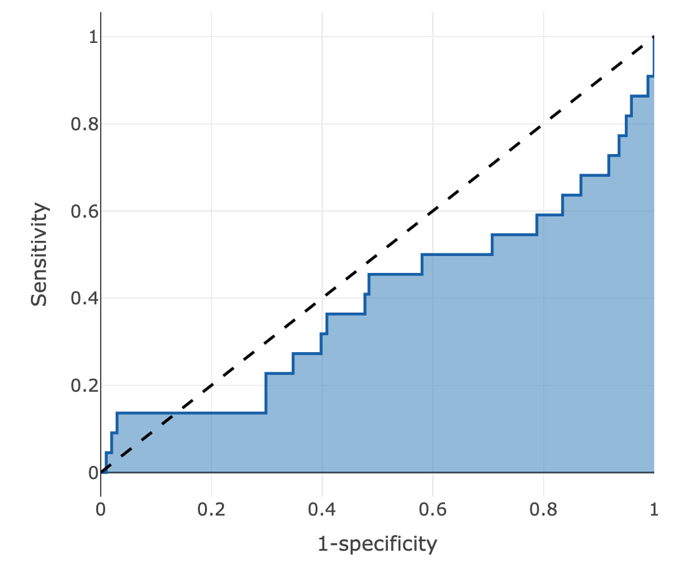 | 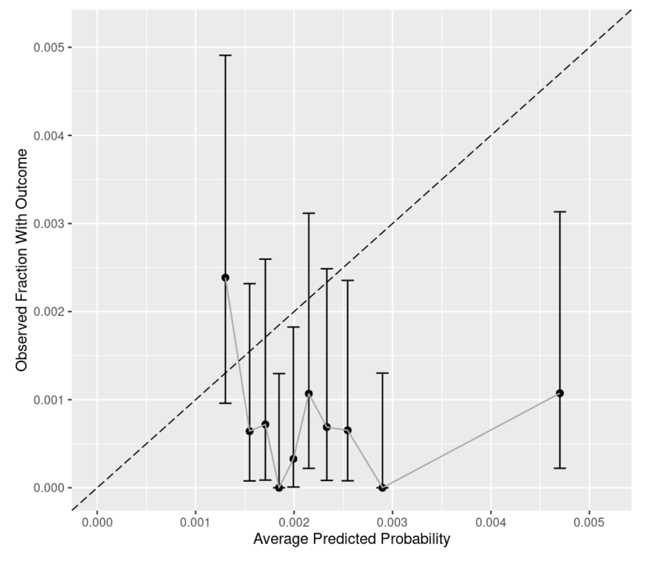 | |
| AU_ePBRN | | |
| 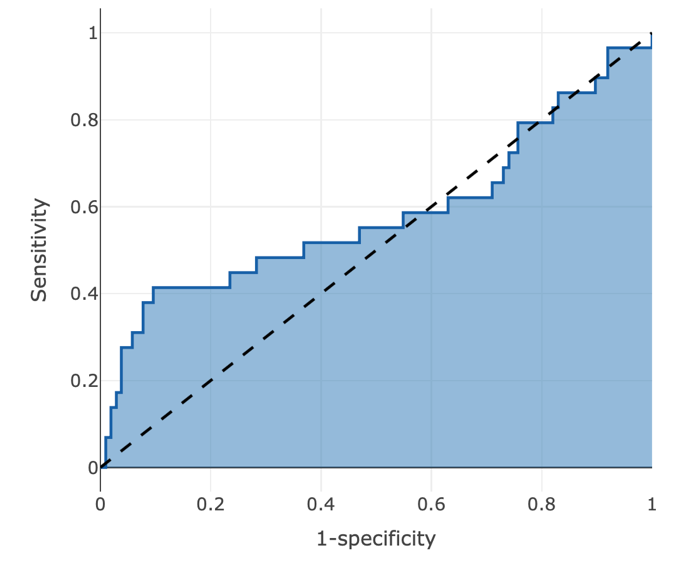 | 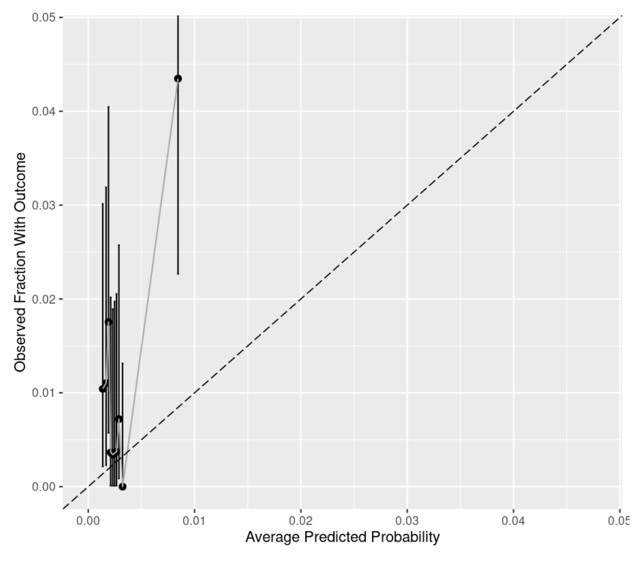 | |
| TRDW | |  |
| 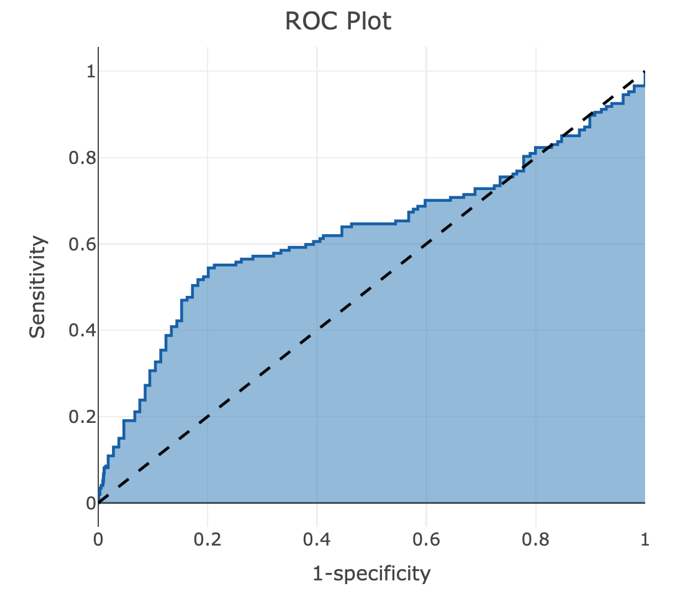 | 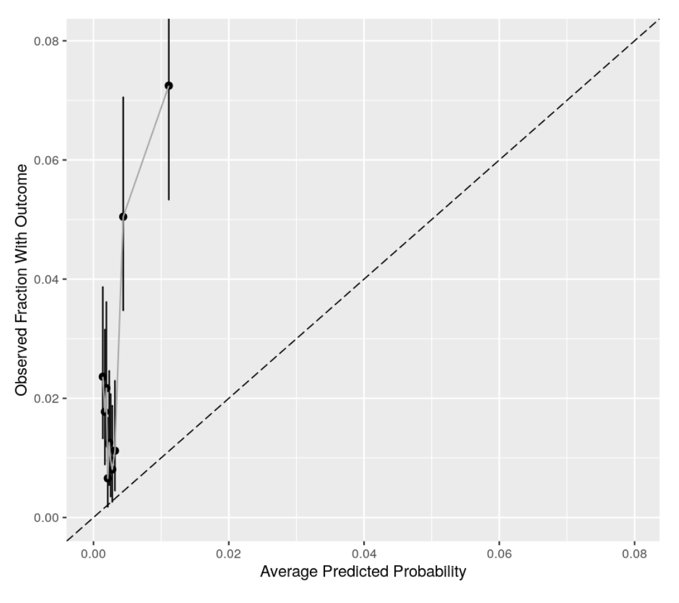 | |
